# Effects of a Physical Exercise Program on Young People and Adults with Autism Spectrum Disorder—A Study Protocol

**DOI:** 10.3390/jcm13195740

**Published:** 2024-09-26

**Authors:** José Pedro Ferreira, Maria João Campos, Sofia Ataíde

**Affiliations:** 1 Faculty of Sport Sciences and Physical Education (FCDEF), University of Coimbra, 3040-248 Coimbra, Portugal; jpferreira@fcdef.uc.pt (J.P.F.); mjcampos@fcdef.uc.pt (M.J.C.); 2 Sport and Physical Activity Research Unit (CIDAF, uid/dtp/04213/2020), Faculty of Sport Sciences and Physical Education, University of Coimbra, 3040-248 Coimbra, Portugal

**Keywords:** autism spectrum disorder, physical exercise, physical fitness, aerobic training, strength training, quality of life

## Abstract

**Background/Objectives**: Physical exercise (PE) has been shown to have positive effects on the symptoms associated with autism spectrum disorder (ASD). However, there is still no consensus on the most appropriate PE intervention model. With this in mind, we developed a program with the aim of determining the effects of PE on physical fitness, with a view to applying it as a potential treatment. **Methods**: Using an experimental methodology, this research work will recruit 18 institutionalized young people and adults who will be allocated to one of two groups, namely (i) the youth training group and (ii) the adult training group, using low-cost materials. Both intervention groups will perform 90 min of training per session, twice a week, for 12 weeks. Evaluations will be carried out at baseline and month 3. The impact of the exercise program will be assessed based on the variables of anthropometry, body composition, cardiovascular response, and cardiorespiratory fitness. **Results**: The results of this study will contribute to the development of more effective strategies, prescription recommendations, and interventions as a guarantee in future programs of better and greater adherence to PE by institutionalized individuals with ASD. **Conclusions**: In addition, we intend to make the PE program available if it promotes positive effects in the target population.

## 1. Introduction

According to the American Psychiatric Association [1], autism spectrum disorder (ASD) is a complex category of neurobiological developmental disorders, typically diagnosed in childhood. According to the Diagnostic and Statistical Manual of Mental Disorders (DSM-5-TR) [1], this disorder is categorized into three dimensions, namely impairments in verbal and non-verbal communication, impairments in social interaction, and the presence of repetitive and stereotyped behaviors, with an estimated prevalence of around 1% in the global population [2].

Recent evidence suggests that motor behaviors are present in more than 87% of individuals diagnosed with ASD [3], making these individuals functionally dependent on caregivers, resulting in low health-related quality of life (QoL) scores compared to their typically developing peers [4]. This population has lower levels of physical activity (PA) compared to their peers of the same age [5,6,7]. A recent study compared the PA levels of adults with ASD with typically developing adults and found that only 10% of participants with ASD reported completing frequent PA, compared to 50% in the neurotypical group [8].

In addition to being inactive, individuals with ASD are a mostly sedentary population [9], not meeting the recommendations for maintaining or improving their health [10]. The most recent guidelines for adults indicate at least 150–300 min of moderate-intensity PA or 75–150 min of vigorous-intensity PA per week. People with ASD should also participate in exercise sessions that focus on aerobic capacity and muscle strength, involving the main muscle groups, at least 2 days a week [11]. As a result of an inactive and sedentary lifestyle, it is clear that this population has a worsened body composition and a reduced aerobic capacity and muscle strength [12,13,14]. One of the possible reasons for these low levels of PA is the existence of barriers that hinder its practice. Among them, we can find factors such as the financial cost and the lack of adapted exercise programs (EPs), both for individuals and for support institutions [15,16]. In addition, the development of these adapted EPs can contribute to reducing the risk of metabolic and cardiovascular diseases, which reduces health costs and promotes physical fitness, functionality, and QoL.

Given the increasing prevalence of ASD and the lifelong nature of this condition, it has become imperative to address the health and well-being needs of this population throughout all stages of life. The transition to adulthood is a critical period, marked by significant changes that include a downward trend in the practice of physical exercise, which is widely documented in the literature [17,18,19]. In addition, physical exercise has been recognized as an effective non-pharmacological intervention to improve not only the physical condition but also the behavioral, social, and emotional aspects of people with ASD. To date, only three systematic reviews have included participants aged between 18 and 30 [19,20,21], highlighting a lack of data and clear guidelines for prescribing exercise programs that meet the specific needs of this population. Grouping young people and adults allows us to investigate how physical exercise can be an effective intervention to mitigate the challenges associated with this transition, promoting the maintenance of an active lifestyle.

The aim of this study was to evaluate the effects of a 12-week PE program on the anthropometry, body composition, cardiovascular response, and cardiorespiratory fitness of institutionalized young people and adults with ASD. The following hypotheses will be defined: (i) the youth group (YG) will display a significant improvement in all the variables assessed after a 12-week physical exercise program; (ii) the adult group (AG) will display a significant improvement in all the variables assessed after a 12-week physical exercise program; and (iii) one of the groups (YG or AG) will display a significantly greater improvement in all the variables assessed compared to the other group, after a 12-week physical exercise program.

## 2. Materials and Methods

### 2.1. Design of the Study

This protocol describes a non-randomized experimental study, following the Consolidated Standards of Reporting Trials statement [22], with the aim of evaluating the effectiveness of two combined physical capacities (aerobic capacity and muscular strength). Participants will be allocated to one of two groups: youth training (YT) or adult training (AT).

**Figure 1:**
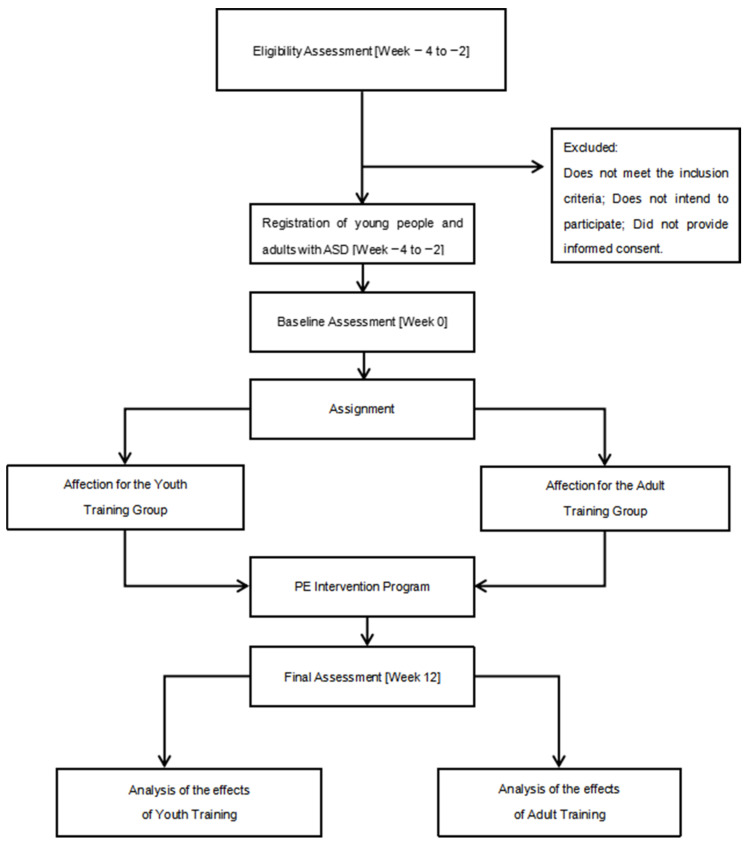
Study design timeline.

Participants will take part in a supervised PE program for 12 weeks, twice a week, for 90 min per session. All outcome measures will be collected at two different times, at baseline (time 0, baseline assessment, week 0) and at the end of the intervention (time 1, final assessment, week 12). Figure 1 shows the study design.

### 2.2. Participants

The program will involve volunteers, comprising young people and adults who are institutionalized in a care facility located in Coimbra (Portugal), recruited using the non-probabilistic convenience method. The YG will include individuals aged 10–17, and the AG will include individuals aged 18–65. This division is based on criteria widely recognized in the literature. Adolescence (10–17 years) is a critical phase of development characterized by rapid physiological and cognitive changes [23]. The adult age group (18–65 years), on the other hand, covers a wide range of stages, from early adulthood to middle age [24]. In the first stage, there will be an individual explanation of the objectives and procedures of the study, as well as the potential benefits, risks, and time required for its development. Secondly, participants/family members/legal guardians will sign an informed consent form.

The inclusion criteria will be as follows: (i) having a certified medical diagnosis of ASD (autistic disorder, Asperger’s syndrome, or pervasive developmental disorder without other specification) according to the standards established in the DSM-5-TR [1]; (ii) ages between 10 and 65 years; (iii) a recent history of non-participation in PE programs or similar physical motor activities; (iv) success in performing movements such as running, pulling, and pushing; (v) ability to perform the assessments; and (vi) attendance at least 75% of the total 24 sessions. The exclusion criteria will be defined as follows: (i) individuals who are unable to commit for 3 months; (ii) individuals with other associated pathologies; (iii) clinical contraindications; (iv) inability to walk without support; (v) inability to communicate; (vi) failure to provide signed informed consent; and (vii) individuals over the age of 18 who do not agree to take part in the study. Specific data on socio-economic status and level of education will not be recorded.

### 2.3. Informed Consent

This research, referenced as CE/FCDEF-UC/00732021, has been submitted to and approved by the Ethics and Research Committee of the Faculty of Sports Sciences and Physical Education of the University of Coimbra (FCDEF-UC). The parents or legal guardians allowing their children to take part in the study will sign a free and informed consent form, which includes all the information about the procedures to be carried out in accordance with the Declaration of Helsinki [10,21]. In this sense, this research is within the norms provided by law for scientific research, with protection for those involved [25,26].

### 2.4. Ethics

Any changes to the protocol will be agreed upon by the research team and formally communicated to the FCDEF-UC ethics committee prior to implementation. Written informed consent will be obtained from the parents or legal guardians (who will be provided with a copy) after the study has been fully clarified (objectives, procedures, expected results, and potential risks). Each participant will then be given a code, guaranteeing their anonymity.

The database and other files inherent to the project will be stored in a secure place using appropriate technical and organizational measures. All the data collected will be used exclusively for this study. Contact with participants will be made in a private environment, and at the end of the research, they will be asked to destroy their “code cards”. In addition, all the information collected will be deleted, except for that which has implications for the conclusion of this project, which will be kept for 5 years according to research policy.

### 2.5. Intervention Assignment and Blindness

Once the initial assessments have been completed, the volunteers will be allocated to the two groups according to their age group. Due to the nature of the intervention, it will not be possible to randomize the sample into groups nor to “blind” participants and the principal investigator.

### 2.6. Protocol

The program is as close as possible to the recommendations suggested by the ACSM [11], as well as the Consort Transparent Reporting Trials [27]. The combined training program can be carried out indoors or outdoors using low-cost materials (elastic bands, balls of different weights, shin pads, mats, ropes, free weights, and steps). It is assumed that not all individuals with or without institutional support can afford a gym membership, and the high financial cost of practicing PA is often one of the barriers pointed out in the literature [16,28,29]. Combined with the need to increase the duration and structure of PE programs for the population with ASD, a 12-week program was designed, with a total of 24 sessions, with the aim of working on the following physical fitness components aimed at health and well-being [11]: aerobic endurance and resistant strength. These sessions have a similar structure to that described in the protocol of the study by Toscano, Carvalho and Ferreira [30]: a preparatory phase, an activation phase, a development phase, and a return to calm. Recent studies indicate that the duration of training programs can be a crucial factor in the success of interventions involving exercise in individuals with ASD. Short-term programs may not provide enough time for physiological and psychological adaptations to occur, which may explain why some individuals do not show significant improvements. Therefore, it is essential to consider that interventions of longer duration may favor a more significant accumulation of physical benefits, such as increased endurance and muscle strength, as well as improvements in mental health, promoting more consistent and sustainable results over time [30].

The weekly frequency of aerobic exercise followed the recommendations of the ACSM [11], with 2 sessions a week for aerobic and muscle strength exercise. This number of weekly sessions favors adaptations that lead to catabolism followed by protein anabolism, allowing lean mass to be maintained or increased [30]. The sessions will last 90 min and will take place twice a week, with recovery periods of at least 48 h, and all interventions will be guided by a physical exercise technician (PET), a psychologist, and four monitors. As far as the type of exercises is concerned, one that focuses directly on the aerobic aspect is prescribed throughout the program and, as far as muscle strength is concerned, a circuit structure is used, recruiting the main muscle groups, taking into account its ease of execution and explanation, simplifying understanding on the part of the individuals. The number of sets varies between 1 and 3, according to the ACSM [11]. Some authors have stated that in untrained individuals, both single sets and multiple sets produce similar increases in upper and lower limb muscle strength, meaning that in the initial stages, strength training, regardless of the number of sets, seems to be effective in improving muscle results [30]. The number of repetitions per set ranges from 8 to 12. The intensity is progressive throughout the program, and the stimuli and loads imposed will be continually adjusted in order to trigger the consequent adaptation processes in the body, taking into account the principle of progressive overload. The program will initially be carried out at a moderate intensity, between 50% and 60% of HRM, progressing to a range of 60%-70% of HRM until reaching a vigorous intensity between 70% and 80% of HRM. In order to monitor the groups’ adherence to the sessions or to suspend the exercise if any participant reports abnormal pain, dizziness, vomiting, or other adverse events, a record sheet will be created to be filled in at each session, representing the only “non-exercise” component utilized during the intervention.

According to Li [31], there are a variety of practical intervention strategies to consider when implementing a PE program for individuals with ASD, as follows: (i) placing participants in a half-moon position whenever important information is provided; (ii) using a short, objective lecture, avoiding wasting useful practice time; (iii) using questioning; (iv) providing collective and individual pedagogical feedback (descriptive, evaluative and prescriptive), both auditory and visual; (v) completing the feedback cycle; (vi) adapting one’s tone of voice to the moment and the practitioner; and (vii) always using demonstrations. The management dimension includes the following strategies: (i) adapting the exercises individually; (ii) drawing up a logical sequence of execution; (iii) defining in advance the order in which the tasks will be carried out; (iv) preparing the material in advance; (v) using few materials; (vi) complying with the predefined timings at each moment of the session; (vii) reducing the transition time between exercises; (viii) asking the participants to help collect the material; and (ix) creating routines. As for the climate dimension, the following practices must be applied: (i) active circulation around the space; (ii) correct positioning in front of the participants; and (iii) an avoidance of dead time, which leads to distractions. Finally, we highlight the discipline dimension with the following practices: (i) reprimanding deviant and incorrect behavior and (ii) using eye contact, posture, and an appropriate image to capture the attention of the practitioners. Table 1 shows the details of the PE program.

## 3. Results

Participants will undergo two assessments, one at the beginning of the PE program and one at the end, consisting of three stages: (i) body composition assessment; (ii) cardiovascular response assessment; and (iii) cardiorespiratory fitness assessment.

### 3.1. Instruments

#### 3.1.1. Anamnesis Form

The anamnesis form to be applied will contain personal identification and health information; a characterization of development and limitations; and a description of the therapeutic path and sporting past [32], which will be completed by the institutional psychologist.

#### 3.1.2. Anthropometric Measurements

Anthropometric measurements will be taken separately in a private room, following standardized procedures [33,34].

##### Height

To measure body mass and height, a scale with a retractable wall stadiometer model 206 (Seca GmbH & Co. KG, Hamburg/Germany) with a gradation of 1 mm. The participant stands barefoot, in a straight position, with the upper limbs alongside the body, feet joined at the heels, and the tips of the feet slightly apart, placing the vertex (upper part of the skull) in the highest position. Subsequently, the body mass index (BMI) will be calculated using the formula

Weight (kg)/Height (m)^2^ (1)

##### Waist Circumference

To measure waist circumference, a 2 m Holtain (Holtain Limited, Crymych/UK) plastic tape measure with an accuracy of 1 mm will be used directly on the skin. Once in the anatomical position, the perimeter will be measured at the midpoint between the lower margin of the tenth rib and the iliac crest [35].

#### 3.1.3. Body Composition

The Inbody 770 (InBody Co., Ltd, Seoul/South Korea), an advanced bioimpedance device that assesses body composition with proven precision, will be used. It is an ideal tool for ongoing research, as it covers an age range of 3 to 99 years, and is also a safe, non-invasive, and fast method. In this sense, it is a technology that uses direct segmental multi-frequency bioimpedance (DSM-BIA) measurement, a tetrapolar system with eight electrodes, which varies the frequency from 1 to 1000 KHz, with an approximate measurement time of 60 s [36].

The following parameters will be obtained from the assessment: total body water; extracellular water; intracellular water; bone mineral content; degree of obesity; body composition history; impedance and reactance of each segment; total and segmental body phase angle; body and segmental edema index; body fat index; fat-free mass index; body cell mass; lean mass of each body segment; estimated segmental fat; skeletal muscle mass; minerals; visceral fat level; body fat percentage; protein; waist-to-hip ratio; and basal metabolic rate.

The participant should climb onto the equipment platform barefoot to establish contact with the four electrodes on their feet. After being prompted by the device, they should take the two electrodes in their hands and move their upper limbs away from their torso, maintaining this position and looking straight ahead.

#### 3.1.4. Functional Profile

##### Childhood Autism Rating Scale

To characterize the profile and level of severity of the participant’s disorder, the Childhood Autism Rating Scale (CARS) instrument will be used [37,38] to be completed by the clinical psychologist who accompanies the individuals in the referral service. This study will use the CARS2-ST to assess the functionality of the sample group up to the age of 16, which was validated and translated into Portuguese by Pereira et al. [39].

This scale evaluates behaviors in 14 domains generally affected by autism spectrum disorder, added to a single category for the description of general impressions [40,41]. The 15 assessment items are as follows: (1) interaction with people; (2) imitation; (3) emotional response; (4) use of the body; (5) use of objects; (6) adaptation to change; (7) reaction to visual and (8) auditory stimuli; (9) response to and use of taste, smell and touch; (10) fear or nervousness; (11) verbal communication; (12) non-verbal communication; (13) activity level; (14) coherence of intellectual response; and, finally, (15) general impressions. The score assigned to each domain ranges from 1 (within normal limits) to 4 (severe autistic symptoms). The total score ranges from 15 to 60, and the cut-off point for ASD is 30 [42].

##### Autism Spectrum Quotient (ASQ)

In order to measure functionality in the remaining group (adults aged 16 and over), the Portuguese version of the Autism Spectrum Quotient (AQ) form will be used, adapted by São Luís Castro and César Lima at the Speech Laboratory of the Faculty of Psychology and Educational Sciences of the University of Porto [43].

The original version of the AQ [44] is simple to apply and provides a measure to quantify the presence of autistic traits. It consists of a questionnaire of 50 statements about five different areas in which pathologies within the disorder have a distinct profile (social skills, attention modulation, attention to detail, communication, and imagination), in which the participant or clinical psychologist is asked to answer whether they totally agree, slightly agree, slightly disagree or totally disagree, with 1 point being given to hypotheses directed towards typically autistic behavior and 0 points to the rest, obtaining a maximum score of 50 points.

#### 3.1.5. Cardiovascular Response

The Omron Digital Blood Pressure Monitor HEM-907 digital sphygmomanometer (Omron Healthcare Europe BV, Matsusaka/Japan) will be used to measure hemodynamic parameters such as systolic blood pressure (SBP), diastolic blood pressure (DBP), and resting heart rate (RHR). Before data collection, participants will remain completely at rest for 5 min, with their legs uncrossed, back and arms supported, without talking and/or moving [45]. Participants will be instructed to sit comfortably in a chair, keep their eyes open, breathe calmly, and avoid movement during data collection. A standard cuff with an inflatable sleeve (12–13 cm wide and 35 cm long) will be used, placed at the level of the heart, with the back and arm properly supported to avoid muscle contraction and BP rises dependent on isometric movement. Two measurements will be taken 1–2 min apart, and the average of these readings will be recorded. If the values differ by ≥10 mmHg, a third measurement will be taken [46]. Measurements will be taken in the morning, and participants will be instructed to avoid caffeine, exercise, and smoking at least 30 min before the measurement [45]. After the test is completed, the data will be downloaded via the Polar Flow Web Service as “.txt” files.

#### 3.1.6. Cardiorespiratory Fitness

##### Six-Minute Walk Test

This is a submaximal test, which aims to assess the distance an individual is able to walk for 6 min, in which two tests are required, with a minimum interval of 30 min between them. The usual medication and walking aids should be maintained. There should be no warm-up period. The individual should sit at rest in a chair, close to the starting position, for at least 10 min before the test begins. During this time, it should be checked whether the individual meets any of the contraindications for taking the test [47]. When it is implemented, the following will be recorded: peripheral oxygen saturation (SpO^2^), SBP, DBP, HHR, perceived dyspnea, and lower limb fatigue (Modified Borg Scale).

##### Chester Step Test (CST)

According to Sykes [48], the CST is a submaximal exercise test in which the individual has to go up and down a step at a controlled speed, according to a series of pre-recorded sound signals, for 10 min.

The CST is incremental, which means that at each step, the speed at which the individual has to go up and down increases (five steps of 2 min each). The metronome sets the cadence of the steps, which starts at step 1 (15 steps per minute) and progresses to step 2 (20 steps per minute), step 3 (25 steps per minute), step 4 (30 steps per minute), and step 5 (35 steps per minute). The choice of step height depends on the participant’s age, functional capacity, BP level, and body composition, ranging from 15 cm to 20 cm, 25 cm, and 30 cm. A height should be selected that allows the individual to comfortably reach level 3 of the test.

Two tests will be carried out by the same assessor (one experimental), with an interval of at least 30 min between them. HR will be recorded every two minutes using the Polar HR sensor. The perception of fatigue and SpO^2^ will be recorded at the end of each level, using the Modified Borg Scale and a MYCO OXI-200A finger oximeter, respectively. The CST ends when the participant reaches 80% of the reserve HR, using the equation (220-age) [49], if the SpO^2^ is below 85% or if the participant is unable to maintain the cadence of steps for 15 s. The test should be suspended if the participant’s perceived exertion is higher than 7, if they decrease their frequency as the level increases, and, after receiving positive reinforcement, do not react. If 80% of the reserve HR is reached halfway through a level, as long as there are no signs of discomfort, the test should continue.

#### 3.1.7. Heart Rate Monitoring

POLAR^®^ model V800 (Polar Electro Oy, Oulu/Finland) transmitters and cardiofrequency meters will be used to monitor HR. The cardiofrequency meter will be programmed to continuously monitor HR, placed on the chest, roughly at the level of the xiphoid apophysis of the sternum, and adjusted so that it does not fall off and so that it does not become uncomfortable during breathing. To facilitate data transmission, the sensor that is in contact with the skin should be covered with a small amount of gel beforehand. The receiver will be placed on the subject’s left wrist. Fifteen minutes before the start of the test, the signal will be received, and the resting HR will be recorded. The data from the HR recordings will then be transferred to the computer via an infrared interface.

The prescription of exercise intensity should be based on direct measurements of maximum HR, if possible, since an equation may not predict the true maximum HR in some individuals, in specific populations, or for certain types of exercise [50]. However, in individuals with ASD, direct measurements are difficult to obtain due to the behavioral characteristics of the population [30]. Therefore, in the present study, exercise intensity will be predicted indirectly using the following equation [51]:

Maximum HR = 208 − (0.7 × age in years)(2)

The present study will use a methodology for managing the intensity of the effort, in which the exerciser with ASD performs physical effort in a range that oscillates between 50% and 80% of the maximum HR.

#### 3.1.8. Modified Borg Scale (MBS)

This scale is particularly useful in maximal/submaximal aerobic tests and exercise sessions. The MBS is a 10-point scale (0–10) which helps one to understand the intensity/severity of tiredness in less than 1 min [52]. During its application, the printed MBS (minimum font size 20) will be presented to the individual, and they will be asked to read all the possible levels. It will be explained that 0 means no fatigue at that moment, and 10 means the worst fatigue. The individual will then be asked to rate their feeling of fatigue at that moment based on the scale.

#### 3.1.9. Procedures

The intervention study involves seven phases: (i) The first is the design of the PE program. (ii) The second is the promotion of the [+ Life] program, where a poster will be distributed alluding to the importance of the practice of PA by the population under study, identifying the objectives under research, to be distributed by themselves/families/professionals who deal with this issue. A logo will also be created alluding to the whole process, making it the program’s brand image. (iii) The sample will be recruited: participants/parents/guardians will sign an informed consent form, and the entire project will be carried out in accordance with the Declaration of Helsinki [17,18]. (iv) The sample will be divided into the youth training group (YTG) and the adult training group (ATG). (v) An initial evaluation of the groups without blinding the evaluator will be conducted. (vi) A final evaluation of the groups without blinding the evaluator will be carried out. (vii) Data analysis will be performed: descriptive, comparison between groups, comparison before/after intervention, and correlations.

#### 3.1.10. Predictable Risks

When starting a PE program, people who usually lead a sedentary lifestyle run the risk of feeling some kind of discomfort, fatigue, muscle pain, or even physical injury. Given that the intensity will be increased gradually, it is expected that muscle pain will be slight and brief, attenuated by the habit of stretching the muscles. Injury risk will be mitigated by the direct supervision of the research team in collaboration with professionals from the institution to which the sample belongs. In the event of physical injury, the necessary care will have to be provided to the participant, insurance will have to be taken out, and their inclusion in the project will depend on the consideration of all related parties.

Another inconvenience inherent to the intervention procedures may result in the appearance of symptoms associated with stress [30]. Such risks will be controlled or mitigated with the approach of the research team, in collaboration with the institution’s professionals, since the participants are already familiar with them. If necessary, a preferably playful approach will have to be taken to control the symptoms.

#### 3.1.11. Statistical Analysis

Descriptive parameters such as the mean and standard deviation will be used in the statistical analysis. The normality of the data will be checked using the Shapiro–Wilk test. Significant differences between groups will be analyzed using the Kruskal–Wallis test, while differences between assessment times will be investigated using the Wilcoxon test. The choice of non-parametric statistics is based on the central limit theorem, which suggests that in order to obtain a normal distribution, a minimum sample size of 30 participants is required [53]. The magnitudes of the differences will be assessed by Cohen’s d effect size [54]. In addition, associations between study variables will be checked using Spearman’s correlation coefficient [55]. The significance level adopted for all analyses will be *p* < 0.05. The data will be processed using the Statistical Package for the Social Sciences (SPSS) software, version 28, Chicago, IL, USA.

## 4. Discussion

This pilot study aims to evaluate the effects of a 12-week physical exercise program on anthropometric variables, body composition, cardiovascular response, and cardiorespiratory fitness in young people and adults with ASD. The results are expected to confirm the hypothesis that physical exercise provides significant physiological benefits based on adaptations in the cardiovascular and musculoskeletal systems, contributing to an overall increase in exercise capacity. These results should reinforce the use of exercise as an effective tool in the prevention and treatment of cardiometabolic diseases, the promotion of physical fitness, the improvement of performance in activities of daily living, and the reduction in restrictive and repetitive behaviors often observed in individuals with ASD.

The pilot study that will follow is essential to understand whether this exercise program is effective in reducing the barriers that hinder the practice of physical exercise by young people and adults with ASD, taking into account the low levels of physical fitness of this population [5,6,7], revealing a strong multidisciplinary approach, since it is prudent to evaluate the combined effects of exercise with independent variables. The exercise program will be supported by a quarterly plan, which includes the number of sessions, the didactic function of each session, the distribution of content per session, and the list of materials.

As with all studies, ours has some limitations, among which we highlight the following: (i) due to logistical constraints, it will not be possible to randomize the groups; (ii) due to limitations in recruiting participants, it will not be possible to implement a control group and (iii) it will not be possible to control the physical activities carried out outside the exercise program, which could positively or negatively influence the variables studied.

We intend to contribute with implications for practice with new interventions with physical exercise, prescription, and effective strategies, which we believe can contribute, at all levels, to the well-being of individuals with ASD. If the program proves to be effective, it could be implemented in institutions, schools, or clinics specializing in the prevention and treatment of symptoms in young people and adults with ASD, resulting in a tool to improve health-related QoL, but it will also promote greater inclusion and participation in regular physical activities, contributing to the general well-being of this population.

## 5. Conclusions

Just as important as the strategies to be implemented when carrying out training programs is the investment that the physical exercise technician (PET) will have to make in order to have a close relationship with individuals with ASD [56]. A relationship based on friendship and mutual respect will lead to the participant trusting the tasks proposed to them, accepting them, and facing them with greater motivation and enjoyment, which will facilitate the path taken and the achievement of objectives. The PET will have to get to know each participant well; they should have the opportunity to observe them in different contexts of life, getting to know their experiences and circumstances. At times of greater resistance, if this relationship is not cemented, it will be difficult to fulfill the training plan prescribed for the session. It is in the PET’s best interest, in addition to specific knowledge of adapted physical activity, to consider particular approaches to populations with neuro-atypical development.

## Figures and Tables

**Table 1:** Physical Exercise Program [+ Life].

1st Month—Level I										
**Objectives—Exploring the different body segments, ranges of motion, and planes of movement; identifying weight distribution across supports; controlling breathing patterns in aerobic and strength exercises.**										
Walk—Line up and walk around the pavilion.				Duration	Interval	Speed	Intensity	Repetition	Series	Volume
				10 min	1 min	Walk	50–60% HR Max			10 min
Upper Limb Strength		Trunk Strength	Lower Limb Strength							
1st Circuit	UL lateral raise	Abdominal crunch	Stand up and sit down	20 min	1 min	Maximum Concentric Eccentric 3 s	Modified Borg Scale (0–10)	8	1	60 min
2nd Circuit	Flexion on an inclined plane (wall)	Pelvic lift	Hip abduction	20 min	1 min					
3rd Circuit	Flexion on an inclined plane (foam step)	Worm	Unilateral knee extension	20 min	1 min					
4 static stretches				30 s				1	1	1.5 min
2nd Month—Level II										
**Objectives—Emphasize unilateral strength work and symmetry between sides; strengthen the muscles of the upper and lower limbs; improve breathing control in higher-intensity exercises.**										
Walk/Run—In a line, run around the pavilion and then walk around the pavilion until you have completed the time set for the exercise.				Duration	Interval	Speed	Intensity	Repetition	Series	Volume
				10 min	1 min	Walk and Run	60–70% HR Max			10 min
Upper Limb Strength		Trunk Strength	Lower Limb Strength							
1st Circuit	Lateral elevation of UL with a moderate-resistance elastic band	Butterfly sit-ups	Stand up and sit down unipodally	20 min	1 min	Maximum Concentric Eccentric 3 s	Modified Borg Scale (0–10)	10	2	80 min
2nd Circuit	Bicep curls with a moderate-resistance elastic band	Pelvic lift with unipodal support	Hip abduction with shin guards (1 kg)	20 min	1 min					
3rd Circuit	Deadlift with bar	Plank with UL elevation	Unilateral knee extension with shin pads (1 kg)	20 min	1 min					
4th Circuit	UL flexion and extension with a disk (0.5 Kg)	Trunk extension	Step up and down (15 cm)	20 min	1 min					
4 static stretches				30s				1	1	1.5 min
3rd Month—Level III										
**Objectives—Progressively increase range of motion; increase cardiorespiratory capacity and muscular endurance; monitor and adjust breathing patterns.**										
Run—Line up and run around the pavilion.				Duration	Interval	Speed	Intensity	Repetition	Series	Volume
				10 min	1 min	Run	70–80% HR Max			10 min
Upper Limb Strength		Trunk Strength	Lower Limb Strength							
1st Circuit	UL rotation (front and back)	Basic jumping jacks	Lateral shift	15 min	1 min	Maximum Concentric Eccentric 3 s	Modified Borg Scale (0–10)	12	3	75 min
2nd Circuit	Lateral elevation of upper limbs with a high-resistance elastic band	Butterfly sit-ups	Static balance on a unipodal base	15 min	1 min					
3rd Circuit	Bicep curls with a high-resistance elastic band	Pelvic lift with unipodal support	Hip abduction with shin guards (2 kg)	15 min	1 min					
4th Circuit	Rope tsunami	Trunk extension	Unilateral knee extension with shin guards (2 kg)	15 min	1 min					
5th Circuit	UL flexion and extension with a disk (2.5 Kg)	Trunk extension with a ball (3 kg)	Step up and down (20 cm)	15 min	1 min					
4 static stretches				30 s				1	1	1.5 min
Static Stretching										
Upper Limb Strength		Trunk Strength	Lower Limb Strength	Duration		Series	Volume		Recovery	
While standing, interlace your toes and raise your upper limbs: above your head, in the midline of your shoulder and behind your back, in full extension, without ever lifting your feet off the ground.		Lying face down, place your hands under your body on the midline of your chest and lift your upper body up through them.	While standing, raise one of your legs to your chest; hold your ankle next to your buttock, placing your knee towards the ground, and flex your torso over your legs.	30 s		1	1.5 min		None	

HR Max—maximum heart rate; Kg—kilograms; min—minutes; s—seconds; %—percentage.

## Data Availability

The original contributions presented in the study are included in the article. Further questions can be addressed to the corresponding author.
